# Bilateral Fronto-Parietal Integrity in Young Chronic Cigarette Smokers: A Diffusion Tensor Imaging Study

**DOI:** 10.1371/journal.pone.0026460

**Published:** 2011-11-01

**Authors:** Yanhui Liao, Jinsong Tang, Qijian Deng, Yongwen Deng, Tao Luo, Xuyi Wang, Hongxian Chen, Tieqiao Liu, Xiaogang Chen, Arthur L. Brody, Wei Hao

**Affiliations:** 1 Mental Health Institute, The Second Xiangya Hospital, Central South University, Changsha, People's Republic of China; 2 Department of Neurosurgery, People's Hospital of Hunan Province, Changsha, People's Republic of China; 3 Department of Psychiatry and Biobehavioral Sciences, University of California Los Angeles School of Medicine, Los Angeles, California, United States of America; 4 Departments of Psychiatry and Research, Greater Los Angeles VA Healthcare System, Los Angeles, California, United States of America; Chiba University Center for Forensic Mental Health, Japan

## Abstract

**Background:**

Cigarette smoking continues to be the leading cause of preventable morbidity and mortality in China and other countries. Previous studies have demonstrated gray matter loss in chronic smokers. However, only a few studies assessed the changes of white matter integrity in this group. Based on those previous reports of alterations in white matter integrity in smokers, the aim of this study was to examine the alteration of white matter integrity in a large, well-matched sample of chronic smokers and non-smokers.

**Methodology/Principal Findings:**

Using in vivo diffusion tensor imaging (DTI) to measure the differences of whole-brain white matter integrity between 44 chronic smoking subjects (mean age, 28.0±5.6 years) and 44 healthy age- and sex-matched comparison non-smoking volunteers (mean age, 26.3±5.8 years). DTI was performed on a 3-Tesla Siemens scanner (Allegra; Siemens Medical System). The data revealed that smokers had higher fractional anisotropy (FA) than healthy non-smokers in almost symmetrically bilateral fronto-parietal tracts consisting of a major white matter pathway, the superior longitudinal fasciculus (SLF).

**Conclusion/Significance:**

We found the almost symmetrically bilateral fronto-parietal whiter matter changes in a relatively large sample of chronic smokers. These findings support the hypothesis that chronic cigarette smoking involves alterations of bilateral fronto-parietal connectivity.

## Introduction

Despite having a comprehensive tobacco control policy, cigarette smoking continues to be the leading cause of preventable morbidity and mortality in China [1] and other developing countries [2], as it already is in developed countries today, and accounts for 5 million deaths globally each year [3]. When cigarettes are smoked, a host of harmful chemicals contribute to the deleterious effects [4], [5]. Mounting scientific evidence proves the association between chronic smoking and lung cancer, chronic obstructive pulmonary disease, (and other chronic respiratory diseases), vascular disease, stroke, and peptic ulcer disease, as well as a wide range of other adverse health effects [6]–[8].

Understanding the mechanism of nicotine dependence and developing better therapies to help with smoking cessation is an urgent need. Emerging technologies, such as neuroimaging and genomics, have contributed to new insights into the neuropharmacology of tobacco addiction [9]. There is considerable literature from functional neuroimaging studies assessing the effects of chronic cigarette smoking on brain structure and function [10]–[12]. However, while several studies have examined gray matter differences between smokers and non-smokers [13]–[15], much is less known about the white matter structural changes in brain in chronic cigarette smokers. Using magnetic resonance imaging (MRI) to examine the brain structure and function in chronic cigarette smoker provides a better understanding about the adverse effects of chronic cigarette smoking on brain.

Diffusion tensor imaging (DTI) is a sensitive method to measure microstructural changes by detecting self-diffusion of water molecules caused by Brownian motion and providing parameters of the diffusion tensor, the most commonly used parameter is fractional anisotropy (FA) [16]. FA is a commonly used measure for examining white matter spatial organization and integrity. Increased FA indicates a non-spherical tensor with preferential orientation in a particular direction, while a decreased FA indicates more isotropic diffusion which has been found to be characteristic of disrupted or damaged whiter matter [17]. It has been widely used to identify and quantify white matter abnormalities in psychiatric and neurological diseases, such as schizophrenia [18] (even in early stage of the illness appearing microstructural disruption of whiter matter [19], bipolar disorders [20], posttraumatic stress disorder [21], generalized anxiety disorder [21], pre-symptomatic Huntington's disease [22] and Huntington's disease itself [23], Parkinson's disease [24], Alzheimer's Disease [25], as well as cigarette smoking [26]. Neuroimaging studies facilitate a better understanding of the neurobiological correlates and consequences of both acute cigarette smoking levels and chronic nicotine dependence [11].

A line of study reported the structural differences in brain gray matter between smokers and nonsmokers [13], [15], [26]–[28]. As for white matter abnormalities with cigarette smoking, Gazdzinski et al studied the effects of alcohol and cigarette smoking on brain volumes and reported that chronic cigarette smoking aggravates chronic alcohol-induced regional brain damage in patients with alcohol dependence. Specifically, compared to non-smokers, alcohol-dependent subjects with chronic cigarette smoking exhibited larger temporal white matter volume by using four-group MANOVA [15]. And, in a study most closely related to the one performed here, Paul et al found that chronic smokers (n = 10) showed significantly higher levels of FA in the corpus callosum than nonsmokers; the low Fagerström scores group exhibited significantly higher levels of FA in the body of the corpus callosum than the high Fagerström group and the nonsmokers. [29]. Jacobsen et al reported that prenatal and adolescent exposure to tobacco smoke showed higher FA in anterior cortical white matter; adolescent smoking also showed higher FA in internal capsule [30]. Recently, Xiaochu Zhang et al examined a relatively large sample of smokers (n = 48) and found that the most highly dependent smokers exhibited lower prefrontal FA, which was negatively correlated with Fagerström Test of Nicotine Dependence [26].

In the present study, we examined white matter changes in a relatively large sample of nicotine dependent smokers and non-smokers matched for a number of demographic variables using DTI.

## Methods

### 1 Participants

Eighty-eight subjects (44 smokers and 44 nonsmoking control subjects), 19–39 years of age, were recruited from the local community using advertisements. They were initially screened during a semi-structured telephone interview to assess smoking, medical, psychiatric, medication, and substance use history. Smokers who had smoked 10 cigarettes per day or more during the previous year and had no period of smoking abstinence longer than 3 months in the past year, and met DSM-IV criteria for nicotine dependence were eligible for this study. All smokers self reported no smoking for the 12 hours before scanning. Nicotine patches were provided as needed. Nonsmoking history was defined as having smoked no more than five cigarettes lifetime.

Participants were excluded if they were a minority other than Han Chinese or had: a diagnosis of mental retardation, current or past alcohol or drug abuse/dependence, a current or past central nervous system disease or condition, a medical condition or disease with likely significant central nervous system effects, history of head injury with skull fracture or loss of consciousness greater than 10 min, a physical problem that would render study measures difficult or impossible, any current or previous psychiatric disorder, a family history of a psychotic disorder, current or previous use of electroconvulsive therapy or psychotropic medications, or a positive pregnancy test. A licensed psychiatrist (Y Liao, J Tang and Q Deng) conducted all clinical interviews. The protocol was approved by the university ethics committee (The Second Xiangya Hospital of Central South University Review Board,No. S054, 2008) and the studies were carried out in accordance with the Declaration of Helsinki. Subjects were fully informed about the measurement and MRI scanning in the study. Written informed consent was given by all study participants.

None of the participants reported daily consumption of alcohol, and none reported experiencing social consequences secondary to alcohol use, or any history with difficulty ceasing alcohol intake. All non-smokers in this sample reported no history of smoking behavior in the past.

### 2 DTI Data Acquisition

Diffusion tensor imaging was performed on a 3-Tesla Siemens scanner (Allegra; Siemens Medical System) at the Magnetic Resonance Center of Hunan Provincial People's Hospital. A standard birdcage head coil was used, along with restraining foam pads to minimize head motion and to diminish the sounds of the scanner. Image sequences were acquired by means of diffusion weighted imaging with single-shot echo planar imaging (EPI) in alignment with the anterior–posterior commissural plane. Integral parallel acquisition technique (iPAT) was used with an accelerate factor of 2. The diffusion sensitizing gradients were applied along 30 nonlinear directions (b = 1,000 s/mm^2^), together with an acquisition without diffusion weighting (b = 0 s/mm^2^). The imaging parameters were 45 continuous axial slices with a slice thickness of 3 mm and no gap, field of view = 240×240 mm^2^, TR/TE = 6046/93 ms, acquisition matrix = 128×128. To provide a high resolution anatomical reference for normalization, axial three-dimensional T1-weighted images were obtained with a spoiled gradient recall sequence with the following parameters: slice thickness = 1 mm, Gap = 0 mm, TR = 2000 msec, TE = 3.7 msec, field of view = 256×256 cm, flip angle = 8°, matrix size = 256×256, Slices = 144.

### 3 MRI Data Analysis

Diffusion tensor images were preprocessed using previously published methods [31], [32]. The diffusion data set was pre-aligned to correct for head motion, and the effects of gradient coil eddy currents using software tools from the FMRIB software library (FSL, http://www.fmrib.ox.ac.uk/fsl). After these steps, the diffusion tensor at each voxel was calculated using the FMRIB diffusion toolbox in FSL. The resulting FA images were transformed into Montreal Neurological Institute (MNI) standard space with Statistical Parametric Mapping 5 (SPM5) (Wellcome Department of Cognitive Neurology, London, UK) by means of the following steps: the b = 0 images were co-registered with the structural T1 image for that individual, the same co-registration parameters were applied to the FA maps (in the same space as the b = 0 images), each individual's T1 image was then normalized to the SPM T1 template (in MNI standard space), and the same normalization parameters were then applied to the co-registered FA images. Finally, FA images were smoothed with an 8-mm full-width at half-maximum Gaussian kernel. Then, all images were re-sampled with a final voxel size of 2×2×2 mm^3^. Each FA image was then spatially smoothed by an 8-mm full-width at half the maximum Gaussian kernel in order to decrease spatial noise and compensate for the inexact nature of normalization.

### 4 Statistical Analysis

Between-group tests were performed on diffusion tensor images of FA using a parametric two sample t-test on a voxel-by-voxel basis using SPM5 software. A prior white matter mask from WFU_PickAtlas (http://www.fmri.wfubmc.edu/) was used to restrict the search volume for analysis. Clusters of 100 voxels or more, surviving an uncorrected threshold of p<0.001, were considered significant. For visualization of the regions showing significantly different FA values between the two groups, significant clusters were superimposed onto SPM5's spatially normalized template brain. Fiber tracts corresponding to the clusters were identified with reference to the Johns Hopkins University DTI-based White Matter Atlas (http://cmrm.med.jhmi.edu; [33].

In order to further investigate the clinical association of the significant clusters, region of interest (ROI) analyses was performed. MarsBar 0.41 (http://marsbar.sourceforge.net/) was used to extract ROIs containing all the voxels classified as white matter from spatially normalized and smoothed FA images. Then, mean FA values of the ROI were calculated using log_roi_batch v2.0 (http://www.aimfeld.ch/). Finally, the average FA values of individual clusters were calculated for each subject. A two-sample t-test (SPSS15.0, Chicago) was used to compare these FA values of the clusters between smokers and non-smoking controls. We used P<0.05 as a statistical threshold to search for significant differences. Correlational analysis of FA values with smoking-related factors including age of smoking onset, number of cigarettes smoked per day, years of smoking and smoking cravings were examined using bivariate correlational analysis (P<0.01).

The T1-weighted images were segmented by using VBM5.1 procedures into white matter, gray matter, and CSF (http://dbm.neuro.uni-jena.de/vbm). Then, the white matter volumes were compared between groups by univariate GLM using total brain volume as covariate.

## Results

### 1 Demographic Findings

Demographic findings are shown in Table 1. The smoker and non-smoker groups were well matched for age, gender, ethnicity, handedness, and alcohol use. Smokers smoked an average of 20 cigarettes per day and 10 year of smoking history. As is typically found in China, the healthy non-smokers, however, had a higher education than smokers.

**Table 1:** Demographic characteristics of the smokers and never-smokers studied.

	Smokers	Never-smokers
Demographic variables		
N	44	44
Age, years, mean±SD	28.0 (5.6)	26.3 (5.8)
Range, years	19–39	19–38
Sex (female/male)	8/36 (18.2%)	10/34 (22.7%)
Subjects' education, years, mean±SD	13.1±2.98	15.0±2.6^a^
Handedness, right/left (n)	42/2	43/1
Married	19 (43.18%)	15 (34.09%)
Drinker/never-drinker^b^	31/13	18/26
Age at start of smoking, mean±SD	17.9±4.3	—
Smoking initiation age range (years)	11–30	—
Years smoking, mean±SD	10.4±5.7	—
Range, years	1.5–21	—
Cigarettes per day	20.3±7.7	
Range, cigarettes per day	10–40	—
Smoking cravings^c^	6.41±1.72	—
Range, scores	3–10	—

a significantly different from control group, p<0.01.

b three participants reported drinking more than once a week among smokers and no non-smoking participants reported drinking more than once a week.

c Before MRI acquisition run, participants were asked to rank their craving from 0 (“not at all”) to 10 (“extreme”).

### 2 Diffusion Tensor Imaging of Smoking Subjects

**Figure 1:**
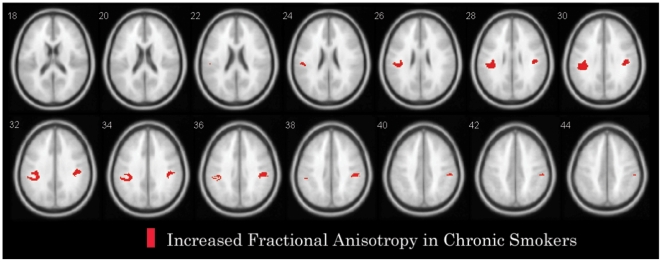
Clusters of increased fractional anisotropy (FA) in smokers compared to healthy non-smokers. Significant FA alterations as revealed by voxel-based whole brain analysis with SPM5 displayed on the FA average map of the sample. Voxels of increased FA in smokers revealed by SPM5 analysis are displayed with red colour. All clusters shown exceed an uncorrected statistical threshold of P<0.001 and a cluster size threshold of 100 consecutive voxels.

Voxelwise analysis revealed increased FA in both the right and left fronto-parietal cortices (superior longitudinal fasciculus) in smokers compared to non-smoking control subjects (Table 2 and Figure 1). In addition, no regions showed significantly decreased FA in smokers compared to the non-smoking control group. There were no significant differences of increased FA values in ROI values between smokers who smoked ≥20 cigarettes/d (n = 12) and those who smoked <20 cigarettes/d (n = 32), between smokers who reported cigarette cravings≥5 scores (n = 28) and <5 scores (n = 16), and between total cigarettes smoked in their lifetime (≥80,000 versus <80,000 cigarettes).

**Table 2:** Group Comparison of Increased Fractional Anisotropy (FA) in White Matter.

Anatomical Region	Right/left	Cluster Size(No. Voxel)	Voxel level p uncorrted	Coordinates (mm)			VoxelZ value
				X	Y	Z	
Parietal, Frontal Cortex	Right	304	0.000	46	−30	38	4.41
Parietal, Frontal Cortex	Left	175	0.000	−40	−30	32	4.08

Uncorrected p<0.001, 100 voxel minimum extent.

### 3 Correlations Between Smoking Related Variables and Diffusion Tensor Imaging Indices of ROI (Region-of-Interest) Regions

For the partial correlational analysis (controlling for age), there were no significant correlations between mean ROI regional FA and smoking associated factors (age of smoking onset, number of cigarettes smoked per day, years of smoking and smoking cravings).

### 4 Effects of education on increased white matter FA values in ROI

Since education levels weren't well-matched between those two groups, correlations between education level (years of education) and increased white matter FA values in the right and left fronto-parietal cortices in the smokers group were examined. There were no significant statistical correlations between education level and increased white matter FA values in the right (Pearson Correlation = −0.208, p = 0.18) and left (Pearson Correlation = −0.076, p = 0.62) fronto-parietal cortices in chronic smoker group.

### 5 white matter volumes in Smoking Subjects

The white matter volumes were compared between groups by univariate GLM using total brain volume as covariate. There is no significant difference between smokers (492.7 ml) and non-smokers (487.2) (F = 2.588, P = 0.11).

## Discussion

The present study provides evidence of microstructural white matter modifications in chronic smokers as measured by whole-brain analysis of FA using DTI. Specifically, increased FA was found in white matter of the bilateral fronto-parietal cortices (superior longitudinal fasciculi) in cigarette smokers relative to healthy non-smoking comparison subjects.

In contrast to the findings here with chronic cigarette smokers, previous studies with other drug dependent subjects revealed decreased FA in white matter of the brain. In patients with heroin dependence, reduced FA was observed in the bilateral frontal sub-gyral cortices, right precentral, and left cingulate gyrus [34]. In cocaine-dependent subjects, lower FA was reported in inferior frontal white matter at the anterior-posterior commissure plane [35], in frontal white matter at the anterior commissure-posterior commissure plane [36], and in the genu and rostral body of the anterior corpus callosum [37]. Similarly, lower FA in the right frontal white matter is also frequently reported in methamphetamine users [38], [39] and alcohol drinkers [40], and recently reported in chronic ketamine users [41]. Convergent evidence suggests that chronic drug use is associated with decreased FA in white matter of the multiple brain regions, especially in the frontal lobe.

Results of this study, along with previous findings [15], [29], [30], suggest increased FA, such that the effects of chronic cigarette smoking on brain white matter are different from effects of other addictive drugs. Increased FA may reflect increased maturation in cell packing density, fiber diameter, and directional coherence [17], [42], [43]. A possible explanation for increased FA could be the variety of neurogenic properties of nicotine. In addition to maintaining and reinforcing smoking behavior, nicotine is reported to have other properties, such as anxiolytic properties [44] and learning and memory-enhancing properties [45]. Despite the proposal that chronic nicotine exposure may ultimately bring no benefits on mood and cognition [46], nicotine per se is known to be a neuroprotective agent, and prevents arachidonic acid-induced injury to neurons and apoptotic cell death [47], [48]. Also, previous studies have revealed that nicotine upregulates calcium-binding proteins [49], increases the levels of intracellular calcium measured [45] and stimulates nerve growth factor [50], which could also be neuroprotective. These previous reported neuroprotective effects could be consistent with increased FA from chronic cigarette smoking.

However, increased FA in white matter of brain in chronic cigarette smokers may not be beneficial. For example, Hoeft et al [51] reported that increased FA of right superior longitudinal fasciculus in Williams syndrome individuals was associated with deficits in visuospatial construction. Similarly, a study [52] of attention deficit hyperactivity disorder (ADHD) also found a correlation between increased FA with deficits in cognitive function. Increased FA is also reported in euthymic bipolar patients [53]. Furthermore, evidence from previous studies [54] reveals that increased FA could be a marker of acute inflammatory processes affecting neural tissue, indicating greater inflammation or less myelination. Thus, our result of increased FA in white matter might be associated with inflammatory changes and axonal damage in fronto-parietal cortex in chronic cigarette smokers. An alternate interpretation for increased FA in chronic cigarette smoking and some psychiatric disorders could be that they reflect the compensatory mechanisms [55] and could be the result of increases in local white matter density.

The higher FA found here is consistent with another study using DTI [29] in 10 chronic cigarette smokers. However, we did not replicate their finding of increased FA in the body and whole corpus callosum in chronic cigarette smokers. Also, a recent study found that both prenatal exposure and adolescent exposure to tobacco smoke were associated with increased FA in anterior cortical white matter [30]. Gazdzinski et al, examined the impact of smoking on alcohol-dependent individuals and found that the combination of cigarette smoking and alcohol dependence results in significantly larger volumes of temporal and frontal white matter [15]; recently, they further confirmed the increased FA result in a abstinent smoking and non-smoking alcoholics study [56]. However, Gons RA et al studied 503 small-vessel disease subjects aged between 50–85 years and found that cigarette smoking is associated with the reduction of FA in cerebral white matter [57]. Age, use of medical drugs and co-morbid medical conditions may the leading cause of the inconsistent results. In our study, increased FA was found in parietal-frontal white matter in the chronic cigarette smokers relative to healthy non-smokers. This discrepancy might arise from sample differences, such as differences in ethnicity, levels of cigarette smoking (smokers in present study had an average of slightly more than 20 cigarettes per day, which were relatively high), age (for example, the mean age in Paul et al, [29] study was 38.5 y and in our study was 28.8 y) and psychiatric comorbidity (such as alcohol dependence).

Results of our study indicate that the maintenance of cigarette smoking might involve fronto-parietal circuitry. Scientific evidence indicaties that the fronto-parietal cortex is one of the crucial units that functionally connects interrelated brain regions [58]. Dosenbach et al [59] indicated that this fronto-parietal circuitry initiates and adjusts control. There is also evidence [60] that there is a network of frontal and parietal areas, which shows significant interactions between changes to a particular stimulus dimension and the current focus of attention. Findings from a previous study suggest that during nicotine withdrawal, functional integration of fronto-parietal networks (involved in verbal working memory) is abnormal in cannabis users [61]. Previous studies and our results may indicate altered connectivity within a cognitive network that is mediated by abnormal neurogenic functional activation (e.g. abnormal cell development) in chronic nicotine exposure. In order to fully understand the mechanism of structural alteration in fronto-parietal cortex of chronic smoking, further studies using techniques such as adaptation or multi-voxel pattern analysis will be needed.

A number of limitations to our study should be addressed. First of all, possible sex differences in the response to nicotine may exist. We did not evaluate sex differences because of the relatively small number of female participants, which is a limitation of the study, although we matched for the gender proportion between smokers and healthy non-smokers. Second, education level was significantly higher in the nonsmoker group compared to the smoker group. However, when we explicitly explored the impact of education level on bilateral fronto-parietal white matter in the smoker group, we found no significant correlation (p>0.1). This suggests that our findings cannot simply be explained in terms of this variable.

In conclusion, our DTI data further support the hypothesis that smokers and non-smokers differed in bilateral fronto-parietal white matter (superior longitudinal fasciculus) integrity. These findings support the hypothesis that chronic cigarette smoking involves alteration of fronto-parietal connectivity.
